# The Kv7 Channel and Cardiovascular Risk Factors

**DOI:** 10.3389/fcvm.2017.00075

**Published:** 2017-12-05

**Authors:** Andreas L. Fosmo, Øyvind B. Skraastad

**Affiliations:** ^1^Division of Physiology, Department of Molecular Medicine, Institute of Basic Medical Sciences, University of Oslo, Oslo, Norway

**Keywords:** Kv7, M-current, KCNQ, cardiovascular disease, hypertension, diabetes, obesity, perivascular adipose tissue

## Abstract

Potassium channels play a pivotal role in the regulation of excitability in cells such as neurons, cardiac myocytes, and vascular smooth muscle cells. The KCNQ (Kv7) family of voltage-activated K^+^ channels hyperpolarizes the cell and stabilizes the membrane potential. Here, we outline how Kv7 channel activity may contribute to the development of the cardiovascular risk factors such as hypertension, diabetes, and obesity. Questions and hypotheses regarding previous and future research have been raised. Alterations in the Kv7 channel may contribute to the development of cardiovascular disease (CVD). Pharmacological modification of Kv7 channels may represent a possible treatment for CVD in the future.

## Introduction

In this review, we focus on the family of the Kv7 channel encoded by the KCNQ genes and explore their possible role in cardiovascular disease (CVD) and the risk factors hypertension (HT), obesity and diabetes.

**Figure 1:**
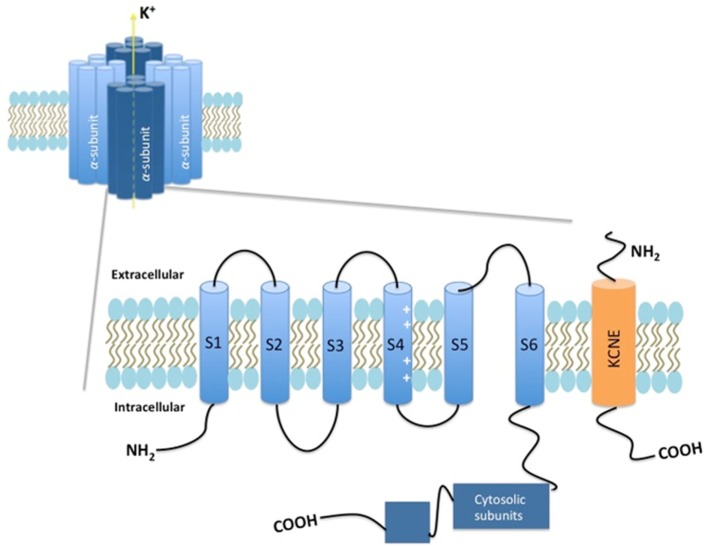
Schematic presentation of the Kv7 channel.

With more than 78 members, the voltage-dependent potassium channels (Kv) are the largest contributor to the superfamily of voltage-dependent ion channels in humans. These channels are encoded by 40 genes and are divided into 12 subfamilies (1). The Kv channels found in mammalians share the same structural property, with tetramers of α subunits. Each of these subunits consists of six transmembrane α-helical structures (S1–S6), with an intracellular localization of both the NH_2_ and COOH termini. The regions S1–S4 constitute the voltage-sensing domain, whereas S5–S6 forms the ion-selective pore. The S4 domain contains four to six positively charged arginines. This charge moves in response to changes in the membrane voltage, in that manner determining the open or closed state of the channel (1–3) (Figure 1).

The Kv7 family has important functions in various tissues throughout the body. As a response to depolarization, these ion channels function as a conductor of K^+^ through the hydrophobic plasma membrane. The chemical gradient of K^+^ is the driving force, which leads to an outward directed current with subsequent hyperpolarization and stabilization of the membrane potential (4). Due to this action, Kv7 channels have a central role in regulating the excitability of neurons, smooth muscle cells, and cardiomyocytes (1).

The Kv7 family comprises five α subunits, Kv7.1–5, encoded by the genes KCNQ 1–5 (5). The α-subunits are arranged as homomers or heteromers (6), each having a characteristic tissue distribution and function (4).

### Regulatory Molecules and Proteins

To function properly, Kv channels depend on endogenous ancillary proteins, which can be separated into cytosolic and transmembrane subunits (4). The transmembrane proteins, called β-subunits, are often essential determinants of the expression and function of Kv channels in general, and there is substantial evidence that this also is the case for Kv7 channels (4, 7, 8). The β-subunits KCNE1 (also called mink) and KCNE2–5 (or mink-related peptides) can associate with the Kv7 channel and regulate its expression and function (5). Best known is the KCNE1 modulation of Kv7.1 in the cardiomyocytes, but KCNE subtypes have been found to exist also in other systems (5). KCNE1 increased Kv7.5 currents by impeding their activation (5). KCNE2 was found to accelerate the activation and deactivation kinetics of Kv7.2/3 currents. KCNE4 enhanced Kv7.4 current amplitude, whereas KCNE3 abolished Kv7.4 and Kv7.5 currents (5).

Cytosolic regulatory molecules and accessory proteins have been shown to interact with the long COOH-terminal region of Kv7 α-subunits (1). The regulatory protein calmodulin seems to play a key role in the Kv7.2/3 maturation and plasma membrane expression (1), the latter possibly due to regulation of the Kv7 channel exit from endoplasmic reticulum (6). In addition, calmodulin suppresses the Kv7 channel as a result of increased intracellular Ca^2+^ concentrations (9). The phosphatidylinositol-(4,5)-bisphosphate (PIP_2_) has an influence on the Kv7 channel open probability, and increased PIP_2_ concentrations stabilize the channel in its open state (10–12). A recent study revealed that both PIP_2_ and βγ G protein should be present for effective Kv7.4 channel function (12). Syntaxin 1A interacts with Kv7.2 and is a possible regulator of neurotransmitter release. The adaptor protein ankyrin-G allows heteromeric Kv7.2/3 to localize to the axon initial segment and Ranvier nodes, important sites for action potential initiation and propagation (1).

Of particular interest is the cytosolic ubiquitin-protein ligase Nedd4-2 that regulates membrane expression of Kv7.2/3 and Kv7.3/5, as well as Kv7.1/KCNE1 heteromultimers. It is believed that Nedd4-2 promotes ubiquitination, internalization, and degradation of the Kv7 channels, thereby downregulating their current (1). A study using HEK-293 cells indicated that the Nedd4-2 pathway might be crucial in regulating the surface density of Kv7 channels in cardiomyocytes and other cell types (13). This mechanism shares some similarities with the enzyme PCSK9, which binds to the low-density lipoprotein receptor (LDL-R) and promotes its lysosomal degradation (14). In the same manner as a PCSK9 inhibitor is used to decrease the degradation of the LDL-R, one might question whether a similar approach would be possible regarding Nedd4-2. A Nedd4-2 inhibitor would theoretically increase the membrane expression of the Kv7 channel and could be considered as a pharmacologically alternative to Kv7 channel openers in the future.

### Tissue Distribution

The Kv7.1–7.5 subunits have a characteristic tissue distribution. Homotetramers of Kv7.1 in cardiomyocytes associate with the ancillary protein KCNE1. Together, they constitute the functional channels that give rise to the slowly activating I_KS_ current (13), which plays a central role in ventricular repolarization (5, 6, 15). Mutations in either KCNQ1 or KCNE1, with a subsequent reduction of the current, may lead to congenital long QT syndrome (13). Kv7.1 has been extensively investigated, and almost 300 mutations in the KCNQ1 gene have been identified. The bulk of these leads to a loss-of-function channelopathy, which in turn promotes long QT syndrome (15). People with such mutations are predisposed to the polymorphic ventricular tachycardia torsades de pointes and sudden cardiac death.

Furthermore, in HEK-293 cells, long QT syndrome-associated Kv7.1 mutants were found to bind far less calmodulin than wild-type Kv7.1 (16). This disturbed calmodulin–Kv7.1 interaction may be crucial for channel expression (1). Overexpression of calmodulin has been shown to increase Kv7.1 expression, whereas impaired calmodulin-binding has been associated with decreased Kv7.1 expression (16). Calmodulin–Kv7.1 interaction may be relevant for the regulation of I_KS_ gating as well. A calmodulin antagonist significantly reduced current density of Kv7.1/KCNE1 in Xenopus oocytes. It therefore seems that an interaction of calmodulin and Kv7.1 is important for the expression and function of I_KS_ and may play a role in the pathophysiology of long QT syndrome. Other consequences of KCNQ1 mutations include altered protein trafficking, with subsequent reduction of cell surface expression, and reduced PIP_2_ sensitivity (15).

The M-current was first discovered in bullfrog sympathetic ganglions (17). Since the initial discovery, the M-current has been found to play a central role in regulating neuron excitability and transmitter release. Furthermore, it modulates the characteristics of the action potentials in neurons (6). The channel is widely distributed in both the central nervous system and in peripheral nerves. The M-current consists of tetramers of 7.2, 7.3, and 7.5 subunits (6). They assemble either as homotetramers, or more frequently, in a heteromeric pattern as either Kv7.2/3 or Kv7.3/5 (6, 18, 19).

There is a well-established relationship between certain mutations in the KCNQ2 and KCNQ3 genes and the development of pathologies such as encephalopathy and epilepsy in humans, with benign familial neonatal seizure being the most common phenotype. The mutations may cause a diminished function of the M-current with subsequent neuronal hyperexcitability and reduced seizure threshold (6).

The Kv7 channels play a central role in the sensation of pain as well. It contributes to the initiation and propagation of the neuronal signals originating from painful sensory stimulation. This is due to their role as a regulator of membrane potential in the subthreshold segment of the action potential (20).

Kv7 channels contribute to cell hyperpolarization and regulation of contractility in various rodent and human blood vessels (21–25). Both KCNQ genes and Kv7 proteins were found in the smooth muscle layer of visceral arteries in humans (25), predominantly consisting of the 7.1, 7.4, and 7.5 α-subunits (5). Especially, the α-subunits 7.4 and 7.5 seem to play an important role as regulators of contractility in vascular smooth muscle cells (VSMC) (21, 25–30). Reduced activity of Kv7 has been suggested to result in increased vascular resistance with subsequent HT (21, 27–29).

The presence of Kv7 channels has also been found in rat and hog coronary blood vessels (27, 28, 31). Kv7 channel inhibitors were found to contract isolated coronary arteries, and Kv7 channel activators proved to be effective coronary vasodilators (27, 28). The latter effect was markedly impaired in spontaneously hypertensive rats (SHR), and similar findings were observed in isolated aorta, mesenteric, and renal arteries (27, 28). The impaired effect of Kv7 channel activators was associated with a reduction in Kv7.4 abundance (28). It has also been shown that Kv7 channels in coronary arteries contribute to the vasodilatory effect of adenosine (28, 31) and to hypoxia-induced vasodilation (31) and reactive hyperemia (28).

The contribution of Kv7 in regulating VSMC in rat left coronary artery has been demonstrated to be higher than in right coronary artery (32), due to a higher expression of Kv7.1 and Kv7.5. Left coronary arteries were found to be more responsive to cyclic AMP (cAMP) and hypoxia than the right coronary arteries (32). This implies that the relaxation mediated by the cAMP pathway or hypoxia was more pronounced in the left coronary arteries than in right coronary arteries.

## Hypertension

The overall prevalence of HT is estimated to be approximately 30–45% in the general population and is more prevalent in the elderly (33). Multiple observation studies have reported that increased blood pressure (BP) is associated with cardiovascular (CV) and renal morbidity, as well as overall mortality (33). HT is by far the most important risk factor for stroke events in humans (33) and estimates indicate that hypertensive heart disease will become one of the leading causes of death globally in 2030 (34).

Hypertension can be divided into primary and secondary HT. In primary HT, also known as essential or idiopathic HT, the underlying pathology is not fully understood. Contrary to this, secondary HT has a clear connection to underlying pathology such as renovascular disease, renal failure, pheochromocytoma, and hyperaldosteronism (35).

### The Role of Kv7 Channels in Autonomic Dysfunction

Hypertension is associated with sympathetic hyperactivity and parasympathetic hypoactivity (36, 37). An increased resting heart rate (HR) may indicate autonomic dysfunction (38). Chronic elevated HR causes persisting pulsatile stress on the arterial wall and results in remodeling and stiffening of the arteries (38). This may lead to a sustained reduction of the arterial diameter, resulting in a rise in total peripheral resistance (TPR). As an indicator of autonomic dysfunction and contributor to hemodynamic stress, elevated HR plays a central role in the stratification of CV risk.

Kv7.2 and Kv7.3 mediate the slow voltage-gated M-current, which plays a crucial role in neuronal excitability and transmitter release (39–41). Autonomic imbalance with an increased HR may be a result of an increased M-current activity located in the efferent parasympathetic ganglion in SHR (39). This may lead to hampered transmitter release, with a reduced vagal control and a subsequent increase in HR (36, 39).

Changes in BP are sensed by the baroreceptors located in the sensory nerve terminals of the carotid sinus and the aortic arch (42). From the aortic arch, the afferent signals are mediated by the aortic depressor nerve, relayed through the nodose ganglia to the nuclei of the nucleus tractus solitarius in the brainstem (43). Increased BP leads to a rise in afferent nerve activity with subsequent decreased efferent sympathetic and increased efferent parasympathetic activity. The inverse response occurs when the BP decreases (44). Immunocytochemical, voltage clamp studies on isolated rat aortic arch demonstrated the presence and function of Kv7.2, Kv7.3, and Kv7.5 in both the nodose ganglia (43, 45) and sensory terminals of aortic baroreceptors (43). The Kv7 channel activator retigabine increased the activation threshold of arterial baroreceptors by nearly 20 mmHg (43). Due to this, one might speculate if the increased M-current activity reduces the baroreceptor sensitivity and result in a subsequent rise in sympathetic activity and BP.

Loss-of-function mutations in KCNQ2 and KCNQ3 are a well-established cause of the benign familial neonatal seizures (6). Besides the symptom of convulsion under a seizure, these patients also present with apnea and bradycardia (45). It may be speculated if the lack of neuronal Kv7 channel function in these patients may cause hyperactivity in the baroreflex and precipitate an increased parasympathetic activity with subsequent bradycardia. One may also speculate if the increased M-current activity seen in the efferent parasympathetic branch of SHR (36) is present in the afferent limb of the baroreflex as well. This may contribute to the decreased pressure control and sympathetic hyperactivity seen in HT.

### Increased Vascular Tone

**Figure 2:**
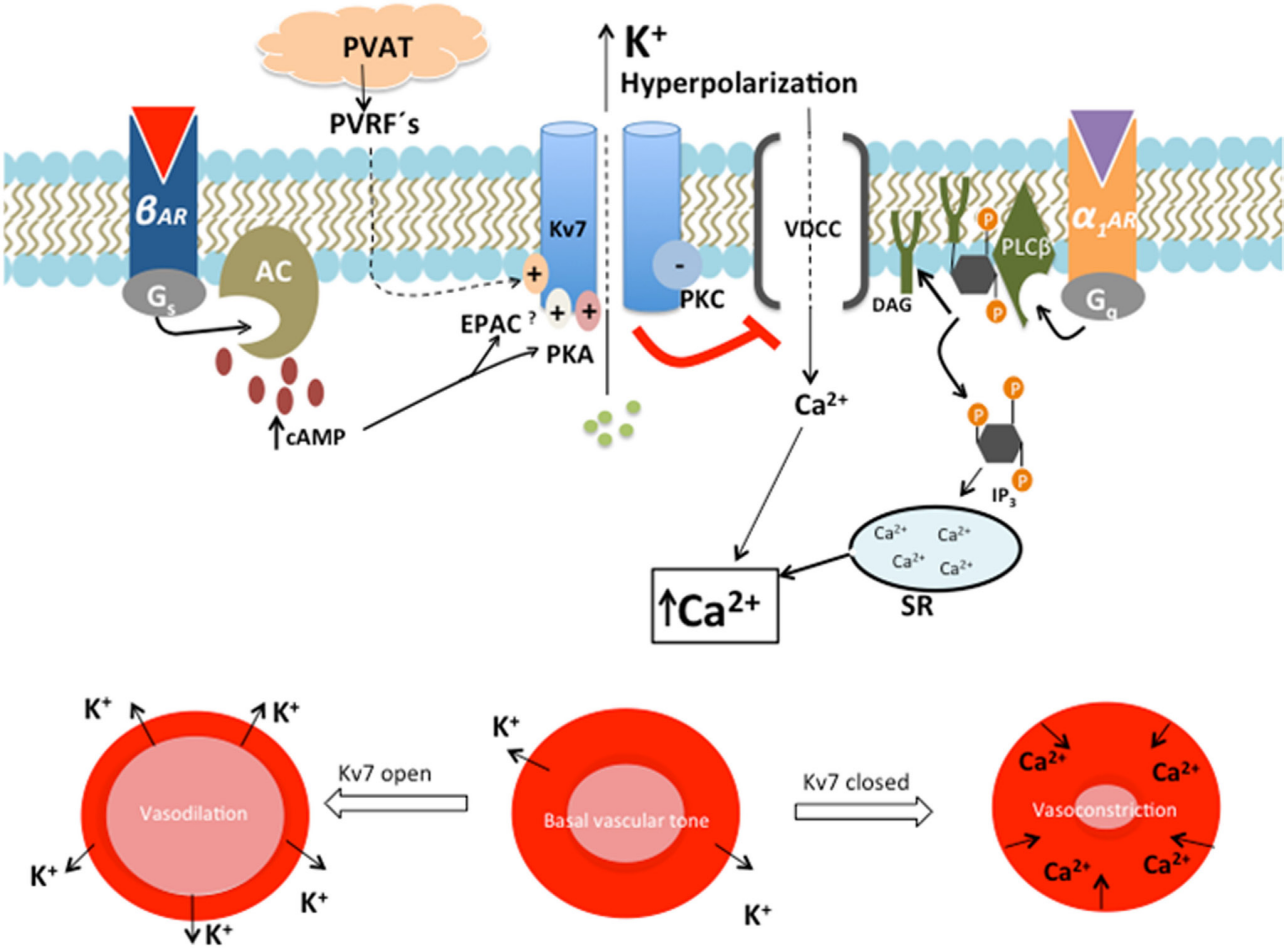
The role of Kv7 in regulating the contractile state of smooth muscle. β-Adrenergic, cyclic AMP (cAMP)-mediated activation of Kv7 leads to outward directed K^+^ current, with subsequent hyperpolarization, reduced opening of voltage-dependent Ca^2+^ channels (VDCC) and vasodilation of the vascular smooth muscle cell. This action opposes the activation of G_q_-linked receptors, α_1_-adrenergic receptor (*α_1_AR*), which negatively regulates Kv7 activity by the actions of the protein kinase C (PKC)-mediated pathway. This decreased Kv7 activity leads to depolarization, which opens VDCC. Ca^2+^ is simultaneously released from sarcoplasmic reticulum (SR) as a response to inositol triphosphate (IP_3_). This rise in intracellular Ca^2+^ leads to vasoconstriction (47). The expression and function of Kv7.4 are impaired in SHR. This results in decreased β-adrenergic, cAMP-mediated Kv7.4 activation, with a shift toward *α_1_AR* activity. Reduced Kv7.4 activity and α_1_-adrenoceptor hyperactivity lead to a rise in intracellular Ca^2+^ concentration and subsequent vasospasm (21). The vasodilatory effect of perivascular relaxing factors (PVRFs) released by perivascular adipose tissue (PVAT) may be mediated through opening of Kv7.

Primary HT is characterized by increased vascular tone and an elevated TPR. Decreased resting membrane potential in VSMC and depolarization enhances the probability of voltage-sensitive Ca^2+^ channels to open. Increased levels of intracellular Ca^2+^ lead to contraction of VSMC (21, 25, 27) (Figure 2). Through outflux of K^+^ and subsequent hyperpolarization, Kv7.4 and Kv7.5 seem to play a central role in the control of vascular tone and TPR (27, 46).

The role and influence of the Kv7 channels in the pathophysiology of essential HT are not fully understood. Recent studies demonstrated that the expression of total Kv7.4 protein is downregulated in different arteries in SHR compared with normotensive rats (21, 27, 28, 46).

Western blot analysis showed that Kv7.4 protein expression was decreased in the thoracic aorta, mesenteric artery, and renal artery in SHR compared with normotensive rats (46). Correlations between decreased Kv7.4 protein expression and a decrease in KCNQ4 mRNA were found only in the aorta. Thus, the total protein expression was not consistently associated with a change in transcription level, suggesting that the Kv7.4 alterations in activity may be explained by a posttranscriptional mechanism. An inverse correlation has been found between microRNA 153 (miR-153) and expression of Kv7.4 protein in SHR (48). miR-153 binds to the 3′ untranslated region of the mRNA and mediates posttranscriptional gene silencing, thereby repressing gene expression (49). Hence, the miR-153 may be responsible for the inconsistency between expression of Kv7.4 proteins and the amount of KNCQ4 mRNA. Remodeling of the vascular wall is another possible consequence of increased miR-153. Transfection with miR-153 into mesenteric arteries of wild-type normotensive rats showed morphological changes after 24 hours. The study showed a significant increase in media/lumen ratio and in average wall thickness, similar to what is expected in SHR (46). It was not clear if this effect resulted from the interference with the expression of Kv7.4 or was due to other mechanisms (48).

### Lack of Response to Endogenous Vasorelaxants

The Kv7 channel has been demonstrated to play a central role in the cAMP-mediated vasodilatory pathway (21, 29) (Figure 2). Increased cAMP is expected to result in enhanced Kv7 activity due to activation of two intracellular effector molecules, namely, protein kinase A (PKA) (21, 50) and exchange protein directly activated by cAMP (EPAC) (51). The β-adrenergic cAMP-mediated vasodilatory pathway is diminished in the vasculature of SHR (Figure 2) (21, 29). Isoprenaline-induced vasodilation of rat renal arteries was reduced in the presence of the pharmacological Kv7.4 blocker linopirdine, as well as in experiments with siRNA-induced knock down targeting Kv7.4 in isolated vessels (21, 52). The ability of isoprenaline to enhance Kv7 channel activity is diminished in SHR, possibly due to the reduced expression or function of Kv7.4. The consequence is a decreased ability to regulate TPR, which is a characteristic of HT (21, 27). A recent study looked at the Kv7 subunit composition and its importance regarding vascular function. The responsiveness of Kv7 to intracellular cAMP/PKA signal activation followed a pattern of Kv7.5 ≫ Kv7.4/7.5 > Kv7.4 (50). This might suggest that Kv7.4 is less PKA dependent and that the observed reduced vasodilatory function in SHR may be due to impairment in the EPAC pathway. This is supported by the fact that mice without Rap1b, a crucial downstream effector molecule in the EPAC pathway, developed HT (53).

Like cAMP, the cyclic GMP (cGMP)-mediated signaling pathway plays a central role in the control of vascular tone (29, 52). Activation of soluble guanylate cyclase by liberated nitric oxide is one out of two mechanisms that increase cGMP in VSMC. The second mechanism is the activation of transmembrane guanylate cyclase-linked natriuretic peptide-receptors, type A or type B (29, 54).

The Kv7 channel was found to be involved in the cGMP-mediated vasodilation of ANP, CNP, or single-nucleotide polymorphism (SNP) in isolated rat aorta (29). The vasodilatory effect was diminished by the Kv7 channel blocker linopirdine. The vasodilatory cGMP pathway is significantly attenuated in SHR, possibly explained by their reduced expression of the Kv7.4 channel (29). This may contribute to the reduced ability of SHR to regulate TPR (29).

### Kv7 Channels and Renin Release in SHR

The renin–angiotensin–aldosterone system (RAAS) plays a constitutional role in the physiology of BP and the pathophysiology of HT with its effects on vascular tone, sodium retention, sympathetic tone, oxidative stress, fibrosis, and inflammation (55). Renin is secreted from the juxtaglomerular apparatus in the renal cortex and is the rate-limiting step in the RAAS cascade (55, 56). Renin is secreted as a response to β-adrenergic stimulation or renal hypoperfusion (56).

Decreased protein expression or function of Kv7.4 in the renal artery of SHR may lead to renal artery stenosis and reduced vasodilatory responses to β-adrenergic stimuli (21). Thus, decreased luminal diameter may lead to renal hypoperfusion and a subsequent activation of the RAAS cascade resulting in systemic effects (21).

Renin is also secreted as a response to low tubular concentration of Na^+^ and Cl^−^. The low tubular salt concentrations are sensed by the macula densa cells located at the end of the cortical ascending limb of the loop of Henle. The furosemide-sensitive Na^+^–K^+^–2Cl^−^ cotransporter (NKCC2) facilitates the ion transport into the cells of macula densa (56), which rely on the electrochemical gradient of Na^+^ as the main driving force. The gradient is provided by the basolateral Na^+^–K^+^ATPase (57). Also Kv7.1 is expressed at the basolateral membrane of the renal epithelial cells in the cortical ascending limb of the loop of Henle. Kv7.1 contributes to the essential recycling of K^+^, which is necessary for proper function of the basolateral Na^+^–K^+^ATPase (58). It may be speculated that a reduced function of Kv7.1 may lead to decreased Na^+^–K^+^ATPase activity, resulting in decreased generation of the electrochemical gradient of Na^+^. This may in turn lead to decreased NKCC2 function and a diminished salt-sensing capacity of the macula densa, causing increased renin release.

To summarize, M-current activity may be increased in the afferent and efferent nerves monitoring and controlling BP. Furthermore, altered Kv7 expression and function could contribute to increased vascular tone and lack of response to endogenous vasorelaxants. Finally, reduced renal Kv7 function may result in increased renin release and subsequent HT.

## Diabetes

Diabetic patients, as well as those with metabolic syndrome, are at increased risk of developing CVD. Diabetes is an independent risk factor for CVD (59) and often accompanied by additional CV risk factors such as high BP and obesity (33). CVD is the leading cause of mortality in diabetic patients (59).

Kv7 channels can be connected to diabetes and CVD in several ways. First, it has been shown that certain variants in the KCNQ1 gene leads to impaired pancreatic β-cell function with increased risk of future type 2 diabetes (60). It has been suggested that these variants are gain-of-function polymorphisms (61). Second, loss-of-function mutations in KCNQ1, as seen in patients with long QT syndrome, have been shown to cause hyperinsulinemia and subsequent hypoglycemia (61). Third, it has been shown that hyperglycemia (HG) in diabetic rats resulted in reduced Kv7 channel activity, expression, and vasodilatory function in the left coronary artery (32).

### KCNQ1 and Type 2 Diabetes

Kv7.1 contributed to the regulation of insulin secretion in pancreatic β-cells (1). A genome-wide association study examined SNP markers in Japanese individuals and discovered that specific SNPs in KCNQ1 were associated with type 2 diabetes (62). These findings were reproduced in samples from Singaporean and Danish populations (62). The expression of KCNQ1 has been found in the human pancreas and in a cultured, insulin-secreting cell line (62), but the role of Kv7.1 in the molecular pathogenesis of type 2 diabetes still remains unclear. However, KCNQ1 overexpression in a MIN6 mouse β-cell line resulted in markedly impaired insulin secretion by glucose, pyruvate, or tolbutamide (63). To examine this further, the association between the different alleles at the SNP rs2237892 within KCNQ1 and type 2 diabetes was analyzed in a Chinese Han population (64). The results showed that the genotypes CT and CC were associated with an enhanced risk of type 2 diabetes. In addition, subjects with the CC genotype had significantly higher systolic BP, prevalence of HT, and macrovascular disease (64). Although these findings need to be verified in a larger population, they supported that KCNQ1 is associated with type 2 diabetes. Inhibition of Kv channels with tetraethylammonium extended action potential duration, suggesting that blockade of Kv channels may work as an effective tool to increase insulin release (61). Further studies must investigate if this is true for Kv7 as well. If this is the case, one may speculate if Kv7 blockers can be used in the treatment of diabetes in the future.

Subunit-specific modulation of KCNQ K^+^-channels by Src tyrosine kinase (Src) may also contribute to the development of type 2 diabetes. In patch clamp studies on Chinese hamster ovary cells and rat sympathetic neurons, Src was found to phosphorylate the Kv7 channel and suppress the M-current (65). An influence of Src on Kv7.1 was indicated by the fact that the non-receptor tyrosine kinase inhibitors GNF-2 and GNF-5 enhanced β-cell survival in an INS-1 rat insulinoma cell line exposed to streptozotocin apoptosis (66). The Src family is a member of the non-receptor tyrosine kinases (65). Thus, the non-receptor tyrosine kinase inhibitor might work through inhibition of Src, and a connection between Kv7.1 and Src is possible.

### KCNQ1 and Hyperinsulinemia

Inhibition of Kv7.1, as well as KCNQ1 knockdown with siRNA, increased exocytosis and insulin secretion in pancreatic β-cells (67). Kv7.1 is located in both cardiomyocytes and pancreatic β-cells, and it was hypothesized that patients with KCNQ1 long QT syndrome may exhibit increased insulin secretion (61). A study involving 14 patients diagnosed with KCNQ1 long QT syndrome showed increased postprandial insulin release in KCNQ1 mutation carriers compared with two control participants. Furthermore, they displayed higher β-cell glucose sensitivity and lower levels of plasma glucose and serum potassium upon oral glucose stimulation. The patients also showed more symptoms of hypoglycemia (61). Lower serum potassium levels were presumably due to insulin activating the sodium potassium ATPase and possibly also due to fecal loss of potassium as reported for KCNQ1 KO mice. The patients displayed hypoglycemia and hypokalemia, both of which may increase the risk of arrhythmias and sudden death. This may further increase the susceptibility for cardiac events in KCNQ1 long QT syndrome patients (61).

### HG and Kv7

An effect of HG on Kv7 channel function in rat coronary arteries has been detected both *in vitro* and *in vivo*. Kv7 channel activity, expression, and function in left coronary artery were reduced when exposed to HG (68–70). Consequently, Kv7 channel modulators were less effective in left coronary arteries from diabetic rats (32). Hypoxia-induced vasodilation in left coronary artery was reduced in streptozotocin-induced diabetic rats (32). It is worth noting that HG also impaired left coronary artery vasodilation induced by the adenylyl cyclase activator forskolin. A reduced Kv7 channel function may contribute to the coronary vascular dysfunction in diabetes and to a reduced defense mechanism against hypoxia. The endothelial dysfunction seen in diabetic male Wistar rats can be prevented by activation of the nuclear receptor superfamily PPARs β/δ (71). The PPAR β/δ agonist GW0742 inhibited the HG-induced Kv7 channel downregulation. The PPAR β/δ target gene PDK4 may participate in the protective effect of PPAR β/δ (71). The HG-induced impairment of Kv7 channel currents, membrane potential, and Kv7 mRNA abundance was reversed by GW0742 (71). In addition, HG was found to contribute to the rise in NADPH oxidase activity in coronary arteries. Also this effect was prevented by GW0742. These results suggest that the HG-induced Kv7 channel downregulation is a result of oxidative modulation.

One might speculate that the discoveries of Morales-Cano et al. (32) also are true for other blood vessels in the body. The result would be increased TPR, increased BP and, hence, increased afterload, which in turn will reduce the cardiac output, leading to a reduced oxygen supply to the myocardium. An increase in myocardium workload leading to increased oxygen consumption may result in ischemic heart disease, especially when combined with a reduced oxygen supply.

It is possible that regulatory mechanisms of the Kv7 channel may involve some sort of Ca^2+^/calmodulin-dependent kinase II (CaMKII) interaction with calmodulin and Kv7.1 or KCNE1 (16). The enzyme CaMKII has essential regulatory functions in the heart and brain. A chronic activation of CaMKII can be pathological and is seen in heart failure. It may induce pathological changes in Ca^2+^ handling directly, gene transcription and ion channels (72). It has been found that diabetes, and the associated HG, causes modification of CaMKII in human, rat, and mice (72). Hence, an activated CaMKII in diabetic patients may cause a pathological CaMKII interaction with Kv7.1 or KCNE1, leading to a subsequent long QT syndrome and cardiac dysfunction. In addition to the interaction between HG and CaMKII, diabetes may induce arrhythmias through HG-induced electrophysiological changes in cardiac progenitor cells, with reduced repair and regeneration of CV tissue (73). Furthermore, exposure to HG is associated with an increased miR-1/133 expression in cardiac progenitor cells (74). An overexpression of miR-1/133 abolished KCNE1 and KCNQ1 expression, thus reducing the I_KS_ with enhanced risk of lethal arrhythmias (74).

Of importance is that KCNQ1 overexpression may lead to decreased levels of insulin and development of type 2 diabetes. Furthermore, patients with loss-of-function mutations in KCNQ1 and long QT syndrome may have an additional risk for cardiac events due to development of hyperinsulinemia with subsequent hypoglycemia and hypokalemia. In addition, HG may reduce Kv7 activity, expression, and function in coronary arteries.

## Obesity and Perivascular Adipose Tissue (PVAT)

Extensive weight gain, particularly when associated with increased visceral adiposity, is a dominant cause of human HT, accounting for 65–75% of the risk of primary HT (75). High BMI is associated with the development of HT, dyslipidemia, insulin resistance, and diabetes mellitus (DM). This may in turn lead to CVD, such as CHD and ischemic stroke (76). Obesity, i.e., BMI ≥ 30, is considered as an independent predictor of clinical CHD (77).

Cardiovascular disease is associated with endothelial dysfunction and subclinical inflammation in obese patients (76). In addition to endothelial dysfunction, PVAT dysfunctions have recently been identified as a new risk factor for CVD (78). PVAT is an essential paracrine organ that participates in the control of peripheral arteriolar tension and thus TPR (78). Adipocyte-derived relaxing factor (ADRF), angiotensin 1–7, H_2_O_2_, methyl palmitate, and adiponectin have recently been proposed as possible candidates for perivascular relaxing factors (PVRFs) released by PVAT (79). The anti-contractile effect of PVAT was absent in high external K^+^ solutions, suggesting that K^+^ channels were critically involved (80). The identity of the K^+^ channel subtype(s) is still a matter of debate. However, Kv7 channels have been suggested to play an important role as downstream targets of ADRF and possibly other PVRFs (Figure 2) (80).

The vasodilatory effect of ADRF was inhibited by XE991 and linopirdine, indicating that opening of Kv7 channels was involved (81). The anti-contractile effect of ADRF may, to some degree, be mediated through, or modulated by, H_2_S (82, 83). Recent data indicated that the anti-contractile effect of H_2_S in isolated rat and mouse aorta was in part due to activation of Kv7 channels since it was almost completely suppressed by the Kv7.1–7.4 channel inhibitor XE991 (84).

An increase in visceral fat leads to adipose dysfunction and subsequent ADRF malfunction (82). The consequence is reduced paracrine control of vascular tone by PVAT, possibly due to diminished H_2_S release. Reduced PVAT control was associated with systemic arterial HT and especially obesity-related HT. Kv7 channel openers may have a therapeutic effect against HT following ADRF-malfunctions (82, 84). The link between H_2_S and Kv7 channels has primarily been derived from the use of XE991 *in vitro* in rat and mouse aortic rings (85). Malfunctions in the PVAT control of arterial tone have been demonstrated in visceral arteries from SHR and obese mice. PVAT mass and function were found to be reduced in SHR compared with normotensive Wistar Kyoto rats (86). The anti-contractile effect of PVAT was reduced in mesenteric arteries of obese mice as well (87). The malfunction of PVAT in SHR and obese mice was to some degree reversed by Kv7 channel openers, i.e., RX0530727, VRX0621238, VRX0621688, and retigabine (81). Although similar studies have not been done on human tissue so far, these findings indicated that opening of Kv7 channels might improve impaired PVAT-mediated vasodilatation in obese-related HT.

Nearly all studies on PVAT regulation of vascular tone are based on open-ring preparations (79). However, a vasodilating action of PVAT was also demonstrated in a perfused, isolated mouse mesenteric vascular bed. Removal of perivascular tissue resulted in increased vasoconstrictor responses to serotonin (87). This study indicated that the anti-contractile effect of PVAT might function through access from outside the vessel. In studies with open-ring preparations, substances released from PVAT may easily diffuse to the luminal side of the vessel through the open ends, which would not occur in an *in vivo* condition (79).

### Aging and Reduced Effect of PVAT

The anti-contractile effect of PVAT is reduced in aging mice (88). Aging is an independent risk factor for diabetes and CVD (79) and is believed to be non-modifiable. It has recently been shown *in vitro* that an antioxidant-treatment with melatonin restored the anti-contractile effect of PVAT in aging mice, indicating that antioxidants may restore PVAT function (88). Interestingly, it has been shown *in vitro* that H_2_S release from PVAT was reduced in aging rats, and this reduction was compensated by an increased cystathionine beta-synthase (CSE) expression (83). CSE located in VSMC is one out of two enzymes, which generate H_2_S in mammalian tissue (83). If this compensation fails to continue, high BP will develop. Given the recent *in vitro* studies indicating that at least some of the anti-contractile effect of H_2_S was due to Kv7 channel activation (84), one may wonder if a Kv7 channel opener may at least to, some degree, reverse HT in the elderly. Selective Kv7.1 inhibitors in isolated mouse mesenteric or renal artery in KCNQ1^+/+^ and KCNQ1^−/−^ mice showed that Kv7.1 did not mediate the anti-contractile effect of PVAT. Kv7.1 was therefore unlikely to participate in the PVAT regulation of vascular tone (89). The same result was found in rat mesenteric arteries. Kv7.2–5 may therefore play a role in ADRF-induced vasodilation. A possible limitation of this study, as pointed out by the authors themselves, is that the KCNE subunits have an impact on the pharmacology of Kv7.1 (90, 91). Different isoforms of the KCNE subunits may explain the drug, tissue, and species variations seen in various studies. The possible clinical implications of the ancillary proteins and subunits interacting with the Kv7 channels may represent an alternative approach to pharmacologically alter Kv7 channel function. It is important to emphasize the fact that the Kv7.1 activator RL-3 was an effective vasorelaxant in male Wistar rat mesenteric arteries (22). The Kv7.1 blockers HMR1556 and L-768,673 reversed the observed R-L3 vasorelaxation entirely, confirming that the effect was due to Kv7.1 modulation. This study showed that although Kv7.1 does not contribute to resting vascular tone, the channel induced considerable vasorelaxation upon stimulation, especially in resistance-like systemic arteries (22).

### Obesity and Arrhythmias

It has previously been found that obese atrial myocytes in guinea pigs displayed shortened action potential duration, as well as a significantly increased I_K_ density, compared with low fat diet controls (92). Furthermore, atrial myocytes exposed to the saturated free fatty acid, palmitic acid, also expressed an increased I_KS_ density in HEK-293 cells (92). It was hypothesized that removal of palmitic acid from a high fat diet may hamper arrhythmic events in obese patient. A recent study reviewed how obesity might alter cardiac ion channel activity (93). It concluded that little is known about the impact of obesity and I_KS_ regarding long QT syndrome and atrial fibrillation. Additional studies are needed to explore this field further.

To summarize, Kv7 channels may play an important role as downstream targets of ADRF and possibly other PVRFs released by PVAT. Obesity and aging are associated with reduced anti-contractile effect of PVAT and subsequent HT. Finally, recent studies have shown a potential connection between obesity, I_KS_ and arrhythmias.

## Kv7 Channels, Associated Subunits, and Autoimmunity

A recent study summarized the current knowledge of autoimmune K^+^ channelopathies (94). Autoantibodies targeting the extracellular loop between S5 and S6 in Kv7 have been detected in patients with dilated cardiomyopathy (95). This was associated with an increase in I_KS_ and a subsequent shortening of the corrected QT interval (QTc), a known cause of ventricular tachyarrhythmia (95). As emphasized by the researchers, this study focused on the antibodies that bind the extracellular loop of Kv7.1. Consequently, one cannot exclude the presence of antibodies targeting other segments of Kv7.1 or other channels or receptors. Further investigations regarding Kv7.1 autoimmunity showed that rabbits immunized with a Kv7-peptide had a significant shorter QTc compared with controls as well as an enhanced risk to ventricular tachyarrhythmias (96). In addition, Kv7.1 immunization hampered dofetilide-induced QTc prolongation and attenuated long QT-related arrhythmias in a rabbit model. It was hypothesized that anti-Kv7.1 antibodies may serve as a cardioprotective treatment of congenital long QT syndrome (96). Contrary to the intracellular localization of both the NH_2_ and COOH termini seen in the Kv7 channel, the associated β-subunits are single transmembrane proteins with an extracellular NH_2_-terminal and a cytosolic COOH terminal (97). The fact that the NH_2_-terminal is located extracellularly could make it susceptible for extracellular manipulation. If autoantibodies against the NH_2_-terminal of the β-subunit would exist, the result could be non-functional Kv7 channels and development of CV risk factors such as HT, diabetes and obesity.

## Limitations

XE991 has been used to determine Kv7 channel activity indirectly in various tissue (5, 28, 32). The specificity of linopirdine and XE991 decrease in a concentration-dependent manner, and it is so far believed that the Kv7 channel blockers have minimal effects on other ion channels at concentrations up to 10 µM (5). The effects of these drugs on other types of K^+^ channels have been described, especially at higher concentrations (24). Regarding Kv7 channel activators, it has also been suggested that retigabine stimulates GABA synthesis and GABA-activated Cl^−^ channels (39), which could influence the interpretation of Kv7 research.

An implication with intravenous administration of Kv7 channel modulators is the lack of control regarding where they constitute their effect. Linopirdine given intravenously caused dose-dependent increases in systemic BP and mesenteric vascular resistance in anesthetized rats, whereas the opposite was true for flupirtine administered through the same route (24). When administrating a drug like this it is not possible to detect whether the observed changes in mesenteric vascular resistance and systemic BP are due to effect on Kv7 channels in the vasculature or in local sympathetic nerves (24). Identification of drugs that act selectively on either neuronal or vascular Kv7 channels would improve our control and precision when manipulating the distinct Kv7 channels.

Selective activation of Kv7 channel subtypes may to some extent secure tissue specificity. Expression systems have been used to determine vascular effects of Kv7 channel modulators. However, this method omits the effect of the KCNE subunits and other ancillary components of the Kv7 channel (24), which may determine the effects of the Kv7 channel modulators *in vivo*. KCNQ subtype expression patterns observed in rat and mouse arteries make it reasonable to anticipate that drugs modulating Kv7.4 and/or Kv7.5 would presumably have vascular effects, whereas drugs modulating Kv7.2/7.3 may have minor direct vascular effects (24). A drug targeting the Kv7.1 subunit with the intention of treating long QT syndrome might affect Kv7.1 subunits in the inner ear or in the gastrointestinal tract (1), which may cause unwanted side effects. A possible solution to this problem could be to dissect the composition of each of the Kv7 channels and look for differences regarding regulatory molecules and proteins.

A recent study concluded that XE991 and linopirdine are state-dependent blockers that prefer the activated subunit of the neuronal Kv7 channel (98). A patch clamp experiment on homomeric Kv7.2 and heteromeric Kv7.2/3 expressed in Chinese hamster ovary cells showed that neither of the Kv7 channel blockers was efficient during resting membrane potential. Inhibition of the Kv7 channels by XE991 was associated with channel activation (98). Hence, the specificity and efficacy of XE991 may depend on both the concentration of the Kv7 channel blocker and the state of the Kv7 channel.

### Dominance of Male Rats and Possible Sex Differences

The majority of published articles regarding Kv7 channels are using male rats and mice (22, 32, 81–84). This is likely to lead to selection bias and thereby not ensuring that the results are representative for the population as a whole. A case in point is that young, female SHR have a lower BP than male SHR (99). CVD has been regarded as a men’s disease for decades, but it is actually more prevalent in postmenopausal women (100). The CV risk factors such as HT and DM are slightly more prevalent in men than in women globally, whereas the prevalence of overweight differs. BMI is highest in men in most high-income countries, while the opposite is true in lower and middle-income countries. There is considerable evidence that diabetes is a more potent risk factor for CHD in women than in men (100). A recent study found essential sex differences in the sensitivity of expression changes in KCNE4 in the vasculature. This may in turn have crucial implications for vascular reactivity and be a contributory factor causing sex-specific susceptibility to CVD (101). Having these sex differences in mind, it seems relevant to question whether the results from research regarding Kv7 channels can be generalized to both men and women.

## Kv7 Channel Modulators in Clinical Use

Kv7 channels are possible targets in the treatment of diseases that involve increased or decreased neuronal activity. Retigabine has been used as an antiepileptic agent, and flupirtine has been found to be effective as an analgesic. The widely used anti-inflammatory drug diclofenac is a Kv7.2/7.3 activator (24). Kv7 channel blockers have been evaluated as potential anti-dementia drugs due to their ability to increase neuronal excitability and thus brain activity. However, a clinical effect has not yet been observed (24).

There is scarce information about the effects of Kv7 channel activators on systemic BP in humans. One of the goals when administrating an antihypertensive agent is a reduction in TPR, which would be an expected effect of a Kv7 channel opener. Even though flupirtine has been used clinically for decades, there has been little focus on associated CV effects of this drug. Two studies conducted on patients over 20 years ago differed regarding the antihypertensive effect of flupirtine (24). Systematic *in vivo* testing of Kv7 channel activators, both in animals and humans, is required to evaluate the antihypertensive effect of these agents. It is also essential to assess possible unfavorable effects representing clinical contraindications.

## Conclusion

Multiple studies indicate a possible connection between the KCNQ Kv channels and HT, diabetes, and obesity, constituting the metabolic syndrome. The Kv7 channels may therefore function as pharmacological targets for prevention and treatment of these conditions. More research is required to enlighten this yet new field. *In vivo* studies in animals and humans are essential, and development of subtype and tissue-selective Kv7 channel modulators is needed.

## Author Contributions

All authors listed have made a substantial, direct, and intellectual contribution to the work and approved it for publication.

## Conflict of Interest Statement

The authors declare that the research was conducted in the absence of any commercial or financial relationships that could be construed as a potential conflict of interest.

## References

[1] Soldovieri MV Miceli F Taglialatela M. Driving with no brakes: molecular pathophysiology of Kv7 potassium channels. Physiology (Bethesda) (2011) 26(5):365–76. 10.1152/physiol.00009.2011 22013194

[2] Wulff H Castle NA Pardo LA. Voltage-gated potassium channels as therapeutic targets. Nat Rev Drug Discov (2009) 8(12):982–1001. 10.1038/nrd2983 19949402 PMC2790170

[3] Borjesson SI Elinder F. Structure, function, and modification of the voltage sensor in voltage-gated ion channels. Cell Biochem Biophys (2008) 52(3):149–74. 10.1007/s12013-008-9032-5 18989792

[4] Abbott GW The KCNE2 K(+) channel regulatory subunit: ubiquitous influence, complex pathobiology. Gene (2015) 569(2):162–72. 10.1016/j.gene.2015.06.061 26123744 PMC4917011

[5] Haick JM Byron KL. Novel treatment strategies for smooth muscle disorders: targeting Kv7 potassium channels. Pharmacol Ther (2016) 165:14–25. 10.1016/j.pharmthera.2016.05.002 27179745

[6] Greene DL Hoshi N. Modulation of Kv7 channels and excitability in the brain. Cell Mol Life Sci (2017) 74(3):495–508. 10.1007/s00018-016-2359-y 27645822 PMC5243414

[7] Abbott GW. KCNE1 and KCNE3: the yin and yang of voltage-gated K(+) channel regulation. Gene (2016) 576(1 Pt 1):1–13. 10.1016/j.gene.2015.09.059 26410412 PMC4917010

[8] Abbott GW. KCNE4 and KCNE5: K(+) channel regulation and cardiac arrhythmogenesis. Gene (2016) 593(2):249–60. 10.1016/j.gene.2016.07.069 27484720 PMC5166581

[9] Hernandez CC Zaika O Tolstykh GP Shapiro MS. Regulation of neural KCNQ channels: signalling pathways, structural motifs and functional implications. J Physiol (2008) 586(7):1811–21. 10.1113/jphysiol.2007.148304 18238808 PMC2375728

[10] Brown DA Hughes SA Marsh SJ Tinker A. Regulation of M(Kv7.2/7.3) channels in neurons by PIP(2) and products of PIP(2) hydrolysis: significance for receptor-mediated inhibition. J Physiol (2007) 582(Pt 3):917–25. 10.1113/jphysiol.2007.132498 17395626 PMC2075249

[11] Li Y Gamper N Hilgemann DW Shapiro MS. Regulation of Kv7 (KCNQ) K+ channel open probability by phosphatidylinositol 4,5-bisphosphate. J Neurosci (2005) 25(43):9825–35. 10.1523/JNEUROSCI.2597-05.2005 16251430 PMC6725574

[12] Povstyan OV Barrese V Stott JB Greenwood IA Synergistic interplay of Gbetagamma and phosphatidylinositol 4,5-bisphosphate dictates Kv7.4 channel activity. Pflugers Arch (2017) 469(2):213–23. 10.1007/s00424-016-1916-4 27981364 PMC5222924

[13] Jespersen T Membrez M Nicolas CS Pitard B Staub O Olesen SP The KCNQ1 potassium channel is down-regulated by ubiquitylating enzymes of the Nedd4/Nedd4-like family. Cardiovasc Res (2007) 74(1):64–74. 10.1016/j.cardiores.2007.01.008 17289006

[14] Dixon DL Trankle C Buckley L Parod E Carbone S Van Tassell BW A review of PCSK9 inhibition and its effects beyond LDL receptors. J Clin Lipidol (2016) 10(5):1073–80. 10.1016/j.jacl.2016.07.004 27678423

[15] Peroz D Rodriguez N Choveau F Baro I Merot J Loussouarn G. Kv7.1 (KCNQ1) properties and channelopathies. J Physiol (2008) 586(7):1785–9. 10.1113/jphysiol.2007.148254 18174212 PMC2375722

[16] Mustroph J Maier LS Wagner S. CaMKII regulation of cardiac K channels. Front Pharmacol (2014) 5:20. 10.3389/fphar.2014.00020 24600393 PMC3930912

[17] Brown DA Adams PR. Muscarinic suppression of a novel voltage-sensitive K+ current in a vertebrate neurone. Nature (1980) 283(5748):673–6. 10.1038/283673a0 6965523

[18] Jentsch TJ Neuronal KCNQ potassium channels: physiology and role in disease. Nat Rev Neurosci (2000) 1(1):21–30. 10.1038/35036198 11252765

[19] Delmas P Brown DA. Pathways modulating neural KCNQ/M (Kv7) potassium channels. Nat Rev Neurosci (2005) 6(11):850–62. 10.1038/nrn1785 16261179

[20] Tsantoulas C. Emerging potassium channel targets for the treatment of pain. Curr Opin Support Palliat Care (2015) 9(2):147–54. 10.1097/SPC.0000000000000131 25872119

[21] Chadha PS Zunke F Zhu HL Davis AJ Jepps TA Olesen SP Reduced KCNQ4-encoded voltage-dependent potassium channel activity underlies impaired beta-adrenoceptor-mediated relaxation of renal arteries in hypertension. Hypertension (2012) 59(4):877–84. 10.1161/HYPERTENSIONAHA.111.187427 22353613

[22] Chadha PS Zunke F Davis AJ Jepps TA Linders JT Schwake M Pharmacological dissection of K(v)7.1 channels in systemic and pulmonary arteries. Br J Pharmacol (2012) 166(4):1377–87. 10.1111/j.1476-5381.2012.01863.x 22251082 PMC3417453

[23] Joshi S Sedivy V Hodyc D Herget J Gurney AM. KCNQ modulators reveal a key role for KCNQ potassium channels in regulating the tone of rat pulmonary artery smooth muscle. J Pharmacol Exp Ther (2009) 329(1):368–76. 10.1124/jpet.108.147785 19151245 PMC2684066

[24] Mackie AR Byron KL. Cardiovascular KCNQ (Kv7) potassium channels: physiological regulators and new targets for therapeutic intervention. Mol Pharmacol (2008) 74(5):1171–9. 10.1124/mol.108.049825 18684841

[25] Ng FL Davis AJ Jepps TA Harhun MI Yeung SY Wan A Expression and function of the K+ channel KCNQ genes in human arteries. Br J Pharmacol (2011) 162(1):42–53. 10.1111/j.1476-5381.2010.01027.x 20840535 PMC3012405

[26] Chadha PS Jepps TA Carr G Stott JB Zhu HL Cole WC Contribution of kv7.4/kv7.5 heteromers to intrinsic and calcitonin gene-related peptide-induced cerebral reactivity. Arterioscler Thromb Vasc Biol (2014) 34(4):887–93. 10.1161/ATVBAHA.114.303405 24558103

[27] Jepps TA Chadha PS Davis AJ Harhun MI Cockerill GW Olesen SP Downregulation of Kv7.4 channel activity in primary and secondary hypertension. Circulation (2011) 124(5):602–11. 10.1161/CIRCULATIONAHA.111.032136 21747056

[28] Khanamiri S Soltysinska E Jepps TA Bentzen BH Chadha PS Schmitt N Contribution of Kv7 channels to basal coronary flow and active response to ischemia. Hypertension (2013) 62(6):1090–7. 10.1161/HYPERTENSIONAHA.113.01244 24082059

[29] Stott JB Barrese V Jepps TA Leighton EV Greenwood IA. Contribution of Kv7 channels to natriuretic peptide mediated vasodilation in normal and hypertensive rats. Hypertension (2015) 65(3):676–82. 10.1161/HYPERTENSIONAHA.114.04373 25547342

[30] Mani BK Brueggemann LI Cribbs LL Byron KL. Activation of vascular KCNQ (Kv7) potassium channels reverses spasmogen-induced constrictor responses in rat basilar artery. Br J Pharmacol (2011) 164(2):237–49. 10.1111/j.1476-5381.2011.01273.x 21323904 PMC3174403

[31] Hedegaard ER Nielsen BD Kun A Hughes AD Kroigaard C Mogensen S KV 7 channels are involved in hypoxia-induced vasodilatation of porcine coronary arteries. Br J Pharmacol (2014) 171(1):69–82. 10.1111/bph.12424 24111896 PMC3874697

[32] Morales-Cano D Moreno L Barreira B Pandolfi R Chamorro V Jimenez R Kv7 channels critically determine coronary artery reactivity: left-right differences and down-regulation by hyperglycaemia. Cardiovasc Res (2015) 106(1):98–108. 10.1093/cvr/cvv020 25616413

[33] Mancia G Fagard R Narkiewicz K Redon J Zanchetti A Bohm M 2013 ESH/ESC practice guidelines for the management of arterial hypertension. Blood Press (2014) 23(1):3–16. 10.3109/08037051.2014.868629 24359485

[34] Mathers CD Loncar D. Projections of global mortality and burden of disease from 2002 to 2030. PLoS Med (2006) 3(11):e442. 10.1371/journal.pmed.0030442 17132052 PMC1664601

[35] Carretero OA Oparil S Essential hypertension. Part I: definition and etiology. Circulation (2000) 101(3):329–35. 10.1161/01.CIR.101.3.329 10645931

[36] Berg T. Voltage-sensitive K(+) channels inhibit parasympathetic ganglion transmission and vagal control of heart rate in hypertensive rats. Front Neurol (2015) 6:260. 10.3389/fneur.2015.00260 26696959 PMC4672051

[37] Esler M. The sympathetic nervous system through the ages: from Thomas Willis to resistant hypertension. Exp Physiol (2011) 96(7):611–22. 10.1113/expphysiol.2011.052332 21551268

[38] Tjugen TB Flaa A Kjeldsen SE The prognostic significance of heart rate for cardiovascular disease and hypertension. Curr Hypertens Rep (2010) 12(3):162–9. 10.1007/s11906-010-0104-8 20431967

[39] Berg T. M-currents (Kv7.2-7.3/KCNQ2-KCNQ3) are responsible for dysfunctional autonomic control in hypertensive rats. Front Physiol (2016) 7:584. 10.3389/fphys.2016.00584 27965589 PMC5126116

[40] Peretz A Sheinin A Yue C Degani-Katzav N Gibor G Nachman R Pre- and postsynaptic activation of M-channels by a novel opener dampens neuronal firing and transmitter release. J Neurophysiol (2007) 97(1):283–95. 10.1152/jn.00634.2006 17050829

[41] Brown DA Passmore GM. Neural KCNQ (Kv7) channels. Br J Pharmacol (2009) 156(8):1185–95. 10.1111/j.1476-5381.2009.00111.x 19298256 PMC2697739

[42] Lau OC Shen B Wong CO Tjong YW Lo CY Wang HC TRPC5 channels participate in pressure-sensing in aortic baroreceptors. Nat Commun (2016) 7:11947. 10.1038/ncomms11947 27411851 PMC4947175

[43] Wladyka CL Feng B Glazebrook PA Schild JH Kunze DL. The KCNQ/M-current modulates arterial baroreceptor function at the sensory terminal in rats. J Physiol (2008) 586(3):795–802. 10.1113/jphysiol.2007.145284 18048450 PMC2375626

[44] Swenne CA. Baroreflex sensitivity: mechanisms and measurement. Neth Heart J (2013) 21(2):58–60. 10.1007/s12471-012-0346-y 23179611 PMC3547418

[45] Wladyka CL Kunze DL. KCNQ/M-currents contribute to the resting membrane potential in rat visceral sensory neurons. J Physiol (2006) 575(Pt 1):175–89. 10.1113/jphysiol.2006.113308 16777937 PMC1819429

[46] Carr G Barrese V Stott JB Povstyan OV Jepps TA Figueiredo HB MicroRNA-153 targeting of KCNQ4 contributes to vascular dysfunction in hypertension. Cardiovasc Res (2016) 112(2):581–9. 10.1093/cvr/cvw177 27389411 PMC5079273

[47] Stott JB Jepps TA Greenwood IA. K(V)7 potassium channels: a new therapeutic target in smooth muscle disorders. Drug Discov Today (2014) 19(4):413–24. 10.1016/j.drudis.2013.12.003 24333708

[48] Maffei A Di Mauro V Catalucci D Lembo G MiR-153/Kv7.4: a novel molecular axis in the regulation of hypertension. Cardiovasc Res (2016) 112(2):530–1. 10.1093/cvr/cvw208 27635057

[49] Arunachalam G Upadhyay R Ding H Triggle CR. MicroRNA signature and cardiovascular dysfunction. J Cardiovasc Pharmacol (2015) 65(5):419–29. 10.1097/FJC.0000000000000178 25384197

[50] Mani BK Robakowski C Brueggemann LI Cribbs LL Tripathi A Majetschak M Kv7.5 potassium channel subunits are the primary targets for PKA-dependent enhancement of vascular smooth muscle Kv7 currents. Mol Pharmacol (2016) 89(3):323–34. 10.1124/mol.115.101758 26700561 PMC4767407

[51] Stott JB Barrese V Greenwood IA. Kv7 channel activation underpins EPAC-dependent relaxations of rat arteries. Arterioscler Thromb Vasc Biol (2016) 36(12):2404–11. 10.1161/ATVBAHA.116.308517 27789473 PMC5467728

[52] Stott JB Greenwood IA Complex role of Kv7 channels in cGMP and cAMP-mediated relaxations. Channels (2015) 9(3):117–8. 10.1080/19336950.2015.1046732 25975669 PMC4594225

[53] Lakshmikanthan S Zieba BJ Ge ZD Momotani K Zheng X Lund H Rap1b in smooth muscle and endothelium is required for maintenance of vascular tone and normal blood pressure. Arterioscler Thromb Vasc Biol (2014) 34(7):1486–94. 10.1161/ATVBAHA.114.303678 24790136 PMC4224284

[54] Ponikowski P Voors AA Anker SD Bueno H Cleland JG Coats AJ 2016 ESC guidelines for the diagnosis and treatment of acute and chronic heart failure. Rev Esp Cardiol (2016) 69(12):1167 10.1016/j.rec.2016.11.005 27894487

[55] Ghazi L Drawz P. Advances in understanding the renin-angiotensin-aldosterone system (RAAS) in blood pressure control and recent pivotal trials of RAAS blockade in heart failure and diabetic nephropathy. F1000Res (2017) 6:297. 10.12688/f1000research.9692.1 28413612 PMC5365219

[56] Peti-Peterdi J Harris RC. Macula densa sensing and signaling mechanisms of renin release. J Am Soc Nephrol (2010) 21(7):1093–6. 10.1681/ASN.2009070759 20360309 PMC4577295

[57] Castrop H Schiessl IM. Physiology and pathophysiology of the renal Na-K-2Cl cotransporter (NKCC2). Am J Physiol Renal Physiol (2014) 307(9):F991–1002. 10.1152/ajprenal.00432.2014 25186299

[58] Hamilton KL Devor DC. Basolateral membrane K+ channels in renal epithelial cells. Am J Physiol Renal Physiol (2012) 302(9):F1069–81. 10.1152/ajprenal.00646.2011 22338089 PMC3362169

[59] Berry C Tardif JC Bourassa MG. Coronary heart disease in patients with diabetes: part I: recent advances in prevention and noninvasive management. J Am Coll Cardiol (2007) 49(6):631–42. 10.1016/j.jacc.2006.09.045 17291928

[60] Jonsson A Isomaa B Tuomi T Taneera J Salehi A Nilsson P A variant in the KCNQ1 gene predicts future type 2 diabetes and mediates impaired insulin secretion. Diabetes (2009) 58(10):2409–13. 10.2337/db09-0246 19584308 PMC2750226

[61] Torekov SS Iepsen E Christiansen M Linneberg A Pedersen O Holst JJ KCNQ1 long QT syndrome patients have hyperinsulinemia and symptomatic hypoglycemia. Diabetes (2014) 63(4):1315–25. 10.2337/db13-1454 24357532

[62] Unoki H Takahashi A Kawaguchi T Hara K Horikoshi M Andersen G SNPs in KCNQ1 are associated with susceptibility to type 2 diabetes in East Asian and European populations. Nat Genet (2008) 40(9):1098–102. 10.1038/ng.208 18711366

[63] Yamagata K Senokuchi T Lu M Takemoto M Fazlul Karim M Go C Voltage-gated K+ channel KCNQ1 regulates insulin secretion in MIN6 beta-cell line. Biochem Biophys Res Commun (2011) 407(3):620–5. 10.1016/j.bbrc.2011.03.083 21426901

[64] Zhang W Wang H Guan X Niu Q Li W. Variant rs2237892 of KCNQ1 is potentially associated with hypertension and macrovascular complications in type 2 diabetes mellitus in a Chinese Han population. Genomics Proteomics Bioinformatics (2015) 13(6):364–70. 10.1016/j.gpb.2015.05.004 26678516 PMC4747647

[65] Gamper N Stockand JD Shapiro MS. Subunit-specific modulation of KCNQ potassium channels by Src tyrosine kinase. J Neurosci (2003) 23(1):84–95. 12514204 10.1523/JNEUROSCI.23-01-00084.2003 PMC6742119

[66] Karunakaran U Park SJ Jun do Y Sim T Park KG Kim MO Non-receptor tyrosine kinase inhibitors enhances beta-cell survival by suppressing the PKCdelta signal transduction pathway in streptozotocin-induced beta-cell apoptosis. Cell Signal (2015) 27(6):1066–74. 10.1016/j.cellsig.2015.01.018 25683919

[67] Rosengren AH Braun M Mahdi T Andersson SA Travers ME Shigeto M Reduced insulin exocytosis in human pancreatic beta-cells with gene variants linked to type 2 diabetes. Diabetes (2012) 61(7):1726–33. 10.2337/db11-1516 22492527 PMC3379663

[68] Bubolz AH Li H Wu Q Liu Y. Enhanced oxidative stress impairs cAMP-mediated dilation by reducing Kv channel function in small coronary arteries of diabetic rats. Am J Physiol Heart Circ Physiol (2005) 289(5):H1873–80. 10.1152/ajpheart.00357.2005 15937095

[69] Li H Chai Q Gutterman DD Liu Y. Elevated glucose impairs cAMP-mediated dilation by reducing Kv channel activity in rat small coronary smooth muscle cells. Am J Physiol Heart Circ Physiol (2003) 285(3):H1213–9. 10.1152/ajpheart.00226.2003 12763748

[70] Liu Y Terata K Rusch NJ Gutterman DD. High glucose impairs voltage-gated K(+) channel current in rat small coronary arteries. Circ Res (2001) 89(2):146–52. 10.1161/hh1401.093294 11463721

[71] Morales-Cano D Moreno L Barreira B Briones AM Pandolfi R Moral-Sanz J Activation of PPARbeta/delta prevents hyperglycaemia-induced impairment of Kv7 channels and cAMP-mediated relaxation in rat coronary arteries. Clin Sci (Lond) (2016) 130(20):1823–36. 10.1042/CS20160141 27413020

[72] Erickson JR Pereira L Wang L Han G Ferguson A Dao K Diabetic hyperglycaemia activates CaMKII and arrhythmias by O-linked glycosylation. Nature (2013) 502(7471):372–6. 10.1038/nature12537 24077098 PMC3801227

[73] Li Y Yang CM Xi Y Wu G Shelat H Gao S MicroRNA-1/133 targeted dysfunction of potassium channels KCNE1 and KCNQ1 in human cardiac progenitor cells with simulated hyperglycemia. Int J Cardiol (2013) 167(3):1076–8. 10.1016/j.ijcard.2012.10.060 23157812

[74] Ding Y Sun X Shan PF. MicroRNAs and cardiovascular disease in diabetes mellitus. Biomed Res Int (2017) 2017:4080364. 10.1155/2017/4080364 28299324 PMC5337313

[75] Hall JE do Carmo JM da Silva AA Wang Z Hall ME. Obesity-induced hypertension: interaction of neurohumoral and renal mechanisms. Circ Res (2015) 116(6):991–1006. 10.1161/CIRCRESAHA.116.305697 25767285 PMC4363087

[76] Bastien M Poirier P Lemieux I Despres JP. Overview of epidemiology and contribution of obesity to cardiovascular disease. Prog Cardiovasc Dis (2014) 56(4):369–81. 10.1016/j.pcad.2013.10.016 24438728

[77] Poirier P Giles TD Bray GA Hong Y Stern JS Pi-Sunyer FX Obesity and cardiovascular disease: pathophysiology, evaluation, and effect of weight loss. Arterioscler Thromb Vasc Biol (2006) 26(5):968–76. 10.1161/01.ATV.0000216787.85457.f3 16627822

[78] Lian X Gollasch M. A clinical perspective: contribution of dysfunctional perivascular adipose tissue (PVAT) to cardiovascular risk. Curr Hypertens Rep (2016) 18(11):82. 10.1007/s11906-016-0692-z 27787838

[79] Gollasch M. Adipose-vascular coupling and potential therapeutics. Annu Rev Pharmacol Toxicol (2017) 57:417–36. 10.1146/annurev-pharmtox-010716-104542 27732801

[80] Tano JY Schleifenbaum J Gollasch M. Perivascular adipose tissue, potassium channels, and vascular dysfunction. Arterioscler Thromb Vasc Biol (2014) 34(9):1827–30. 10.1161/ATVBAHA.114.303032 25012133

[81] Zavaritskaya O Zhuravleva N Schleifenbaum J Gloe T Devermann L Kluge R Role of KCNQ channels in skeletal muscle arteries and periadventitial vascular dysfunction. Hypertension (2013) 61(1):151–9. 10.1161/HYPERTENSIONAHA.112.197566 23184384

[82] Schleifenbaum J Kohn C Voblova N Dubrovska G Zavarirskaya O Gloe T Systemic peripheral artery relaxation by KCNQ channel openers and hydrogen sulfide. J Hypertens (2010) 28(9):1875–82. 10.1097/HJH.0b013e32833c20d5 20577128

[83] Fang L Zhao J Chen Y Ma T Xu G Tang C Hydrogen sulfide derived from periadventitial adipose tissue is a vasodilator. J Hypertens (2009) 27(11):2174–85. 10.1097/HJH.0b013e328330a900 19644389

[84] Kohn C Schleifenbaum J Szijarto IA Marko L Dubrovska G Huang Y Differential effects of cystathionine-gamma-lyase-dependent vasodilatory H2S in periadventitial vasoregulation of rat and mouse aortas. PLoS One (2012) 7(8):e41951 10.1371/journal.pone.0041951 22870268 PMC3411702

[85] Kohn C Dubrovska G Huang Y Gollasch M. Hydrogen sulfide: potent regulator of vascular tone and stimulator of angiogenesis. Int J Biomed Sci (2012) 8(2):81–6. 23675260 PMC3614859

[86] Galvez B de Castro J Herold D Dubrovska G Arribas S Gonzalez MC Perivascular adipose tissue and mesenteric vascular function in spontaneously hypertensive rats. Arterioscler Thromb Vasc Biol (2006) 26(6):1297–302. 10.1161/01.ATV.0000220381.40739.dd 16601235

[87] Fesus G Dubrovska G Gorzelniak K Kluge R Huang Y Luft FC Adiponectin is a novel humoral vasodilator. Cardiovasc Res (2007) 75(4):719–27. 10.1016/j.cardiores.2007.05.025 17617391

[88] Rosei CA De Ciuceis C Rossini C Porteri E Rezzani R Rodella L 7d.10: effects of melatonin on contractile responses in small arteries of ageing mice. J Hypertens (2015) 33(Suppl 1):e103. 10.1097/01.hjh.0000467627.93881.5e26102657

[89] Tsvetkov D Kassmann M Tano JY Chen L Schleifenbaum J Voelkl J Do KV 7.1 channels contribute to control of arterial vascular tone? Br J Pharmacol (2017) 174(2):150–62. 10.1111/bph.13665 28000293 PMC5192887

[90] Melman YF Krummerman A McDonald TV. KCNE regulation of KvLQT1 channels: structure-function correlates. Trends Cardiovasc Med (2002) 12(4):182–7. 10.1016/S1050-1738(02)00158-5 12069759

[91] Melman YF Um SY Krumerman A Kagan A McDonald TV. KCNE1 binds to the KCNQ1 pore to regulate potassium channel activity. Neuron (2004) 42(6):927–37. 10.1016/j.neuron.2004.06.001 15207237

[92] Aromolaran AS Colecraft HM Boutjdir M. High-fat diet-dependent modulation of the delayed rectifier K(+) current in adult guinea pig atrial myocytes. Biochem Biophys Res Commun (2016) 474(3):554–9. 10.1016/j.bbrc.2016.04.113 27130822

[93] Aromolaran AS Boutjdir M. Cardiac ion channel regulation in obesity and the metabolic syndrome: relevance to long QT syndrome and atrial fibrillation. Front Physiol (2017) 8:431. 10.3389/fphys.2017.00431 28680407 PMC5479057

[94] Lazzerini PE Capecchi PL Laghi-Pasini F Boutjdir M. Autoimmune channelopathies as a novel mechanism in cardiac arrhythmias. Nat Rev Cardiol (2017) 14(9):521–35. 10.1038/nrcardio.2017.61 28470179

[95] Li J Seyler C Wiedmann F Schmidt C Schweizer PA Becker R Anti-KCNQ1 K(+) channel autoantibodies increase IKs current and are associated with QT interval shortening in dilated cardiomyopathy. Cardiovasc Res (2013) 98(3):496–503. 10.1093/cvr/cvt046 23447643

[96] Li J Maguy A Duverger JE Vigneault P Comtois P Shi Y Induced KCNQ1 autoimmunity accelerates cardiac repolarization in rabbits: potential significance in arrhythmogenesis and antiarrhythmic therapy. Heart Rhythm (2014) 11(11):2092–100. 10.1016/j.hrthm.2014.07.040 25087487

[97] McCrossan ZA Abbott GW. The MinK-related peptides. Neuropharmacology (2004) 47(6):787–821. 10.1016/j.neuropharm.2004.06.018 15527815

[98] Greene DL Kang S Hoshi N. XE991 and linopirdine are state-dependent inhibitors for Kv7/KCNQ channels that favor activated single subunits. J Pharmacol Exp Ther (2017) 362(1):177–85. 10.1124/jpet.117.241679 28483800 PMC5478917

[99] Berg T Beta- and Alpha2-adrenoceptor control of vascular tension and catecholamine release in female normotensive and spontaneously hypertensive rats. Front Neurol (2017) 8:130 10.3389/fneur.2017.00130 28424658 PMC5380753

[100] Appelman Y van Rijn BB Ten Haaf ME Boersma E Peters SA. Sex differences in cardiovascular risk factors and disease prevention. Atherosclerosis (2015) 241(1):211–8. 10.1016/j.atherosclerosis.2015.01.027 25670232

[101] Abbott GW Jepps TA. Kcne4 deletion sex-dependently alters vascular reactivity. J Vasc Res (2016) 53(3–4):138–48. 10.1159/000449060 27710966 PMC5166573

